# Upregulation of Unc-51-Like Kinase 1 by Nitric Oxide Stabilizes SIRT1, Independent of Autophagy

**DOI:** 10.1371/journal.pone.0116165

**Published:** 2014-12-26

**Authors:** Junhui Xing, Hongtao Liu, Huabing Yang, Rui Chen, Yuguo Chen, Jian Xu

**Affiliations:** 1 Department of Emergency, Qilu Hospital, Shandong University, Jinan, China; 2 Department of Medicine and Harold Hamm Oklahoma Diabetes Center, University of Oklahoma Health Sciences Center, Oklahoma City, Oklahoma, United States of America; 3 Department of Physiology, University of Oklahoma Health Sciences Center, Oklahoma City, Oklahoma, United States of America; University of Illinois College of Medicine, United States of America

## Abstract

SIRT1 is central to the lifespan and vascular health, but undergoes degradation that contributes to several medical conditions, including diabetes. How SIRT1 turnover is regulated remains unclear. However, emerging evidence suggests that endothelial nitric oxide synthase (eNOS) positively regulates SIRT1 protein expression. We recently identified NO as an endogenous inhibitor of 26S proteasome functionality with a cellular reporter system. Here we extended this finding to a novel pathway that regulates SIRT1 protein breakdown. In cycloheximide (CHX)-treated endothelial cells, NONOate, an NO donor, and A23187, an eNOS activator, significantly stabilized SIRT1 protein. Similarly, NO enhanced SIRT1 protein, but not mRNA expression, in CHX-free cells. NO also stabilized an autophagy-related protein unc-51 like kinase (ULK1), but did not restore SIRT1 protein levels in ULK1-siRNA-treated cells or in mouse embryonic fibroblasts (MEF) from *Ulk1^−/−^* mice. This suggests that ULK1 mediated the NO regulation of SIRT1. Furthermore, adenoviral overexpression of ULK1 increased SIRT1 protein expression, while ULK1 siRNA treatment decreased it. Rapamycin-induced autophagy did not mimic these effects, suggesting that the effects of ULK1 were autophagy-independent. Treatment with MG132, a proteasome inhibitor, or siRNA of β-TrCP1, an E3 ligase, prevented SIRT1 reduction induced by ULK1-siRNA. Mechanistically, ULK1 negatively regulated 26S proteasome functionality, which was at least partly mediated by O-linked-GlcNAc transferase (OGT), probably by increased O-GlcNAc modification of proteasomal subunit Rpt2. The NO-ULK1-SIRT1 axis was likely operative in the whole animal: both ULK1 and SIRT1 protein levels were significantly reduced in tissue homogenates in eNOS-knockout mice (lung) and in db/db mice where eNOS is downregulated (lung and heart). Taken together, the results show that NO stabilizes SIRT1 by regulating 26S proteasome functionality through ULK1 and OGT, but not autophagy, in endothelial cells.

## Introduction

Sirtuin-1 (SIRT1) is an NAD^+^-dependent type III histone deacetylase, which represents the most evolutionarily conserved sirtuin among the seven mammalian homologs [1]. It is widely distributed in tissues and has been implicated in the regulation of inflammation, cellular senescence/aging, cellular apoptosis/proliferation, differentiation, metabolism, and other physiopatholocial processes [2]. Evidence supporting these functions largely stems from loss-of-function and gain-of-function animal studies. For example, SIRT1 ablation has been found to promote loss of epigenetic and genomic hematopoietic stem and progenitor cell maintenance under stress conditions [3]. SIRT1 deletion in mouse pancreatic beta cells disrupts glucose sensing, impairing insulin secretion [4]. Deletion in the liver prompts hepatic steatosis [5], [6] and formation of cholesterol gallstones [7]. Endothelial SIRT1 deficiency induces vascular senescence, producing nephrosclerosis [8]. Conversely, transgenic overexpression of SIRT1 produces phenotypes resembling calorie restriction (CR) [9] and protects against high-fat diet-induced metabolic damage [10]. Similarly, activation of SIRT1 mitigates syndromes such as diabetes, neurodegenerative diseases, liver steatosis, bone loss, and inflammation [11]. For example, SIRT1 activation by resveratrol increases survival of mice on a high calorie diet [12]. This beneficial effect is absent when SIRT1 is depleted [13]. SIRT1 has also been reported to improve healthy ageing and to protect against metabolic syndrome-associated cancer [14].

Therefore, it is not surprising that downregulation of SIRT1 contributes to medical conditions, such as metabolic syndrome and diabetes, in mice and humans. In mouse models of obesity, a high-fat diet induces persistent activation of c-Jun N-terminal kinase 1 (JNK1), which enhances SIRT1 degradation in the liver, leading to hepatic steatosis [15]. SIRT1 degradation in adipocytes promotes metabolic dysfunction [16]. Downregulation of SIRT1 in monocytes has been associated with insulin resistance and metabolic syndrome [17]. Loss of SIRT1 expression is associated with tumor progression in colorectal adenocarcinoma [18]. Reduced hepatic SIRT1 in aged mice leads to impaired body homeostasis and inhibition of liver proliferation [19]. The p38 kinase-mediated proteasomal degradation of SIRT1 contributed to the cellular senescence of the articular chondrocytes induced by ionizing radiation [20]. The regulation of SIRT1 protein turnover is poorly defined. Understanding the mechanism is important, as it will provide insights into the modulation of this molecule as a potential treatment for these disorders.

Two important observations may provide a solution to this conundrum. First, increasing evidence in animal models demonstrates that eNOS and/or eNOS-derived NO positively regulate SIRT1 protein expression. For example, when mice are subjected to CR, eNOS expression is increased [21] and is accompanied by increased SIRT1 expression and other changes like increased mitochondrial biogenesis, oxygen consumption, and ATP production. However, this effect is strongly attenuated in eNOS knockout mice, suggesting an essential role of eNOS. Similarly, chronic inhibition of phosphodiesterase (PDE) 5 enhances eNOS-induced SIRT1 signaling in the hearts of diabetic mice [22]. Administration of testosterone increases eNOS activity and restores SIRT1 expression in mouse models of testosterone deficiency [23], similar to the effects of androgen depletion in humans [24]. Cilostazol, a selective inhibitor of PDE3, upregulates SIRT1 protein expression via eNOS-derived NO [25], representing a new pathway in endothelial senescence [26].

Second, we have recently identified that eNOS-derived NO functions as a physiological suppressor of 26S proteasome functionality in vascular endothelial cells, through an O-linked GlcNAc transferase (OGT)-dependent mechanism [27]. As the key component of the ubiquitin proteasome system responsible for regulated degradation of most intracellular proteins [28], [29], 26S proteasomes recognize, unfold, and, ultimately, destroy proteins. Most proteasome-targeted proteins undergo ubiquitination, that is, are tagged with polyubiquitin chains, for degradation [30], [31]. It has been reported that SIRT1 is ubiquitinated in response to persistent activation of JNK1 and thereby targeted for proteasomal degradation [15]. This finding has been confirmed by other studies [16], although the mechanism remains elusive. We hypothesized that NO stabilized SIRT1 protein expression through regulation of 26S proteasome functionality.

In this study, we discovered that unc-51 like kinase 1 (ULK1), an autophagy-related protein, mediated NO regulation of SIRT1, independent of autophagy, through OGT-dependent modulation of 26S proteasome functionality. This study highlights a new regulatory mechanism connecting a gaseous hormone (NO) to a protein (SIRT1) that is central to the lifespan and vascular health.

## Materials and Methods

### Reagents

All antibodies were purchased from Santa Cruz Biotechnology (Santa Cruz, CA), except for the following: mouse-derived antibody against Rpt2 or β7 in the 19S proteasome complex (PA700) from Abcam (Cambridge, MA); anti-O-GlcNAc antibody (CTD110.6 antibody) from Sigma (St Louis, MO), and; anti-eNOS, LC3B, Beclin-1, p62, and β-TrCP1 from Cell Signaling (Danvers, MA). All chemicals were procured from Fisher Scientific (Pittsburgh, PA), except for the following: fluorogenic proteasome substrates from Calbiochem (San Diego, CA); epoxomicin from Sigma (St. Louis, MO); negative control siRNA or target-specific siRNA duplex against human ULK1 and β-TrCP1 from Santa Cruz Biotechnology (Santa Cruz, CA), and; protease inhibitor cocktail from Sigma (St Louis, MO). Adenoviral vectors expressing Green fluorescent protein (GFP) and O-GlcNAc transferase (OGT) were prepared as previously described [32], [33] and kindly provided by Dr. Gerald W. Hart of Johns Hopkins University. Adenoviral vector overexpressing eNOS was prepared as described previously [34] and kindly provided by Dr. Donald D. Heistad of the University of Iowa. Plasmids expressing Ub^G76V^-GFP (Addgene plasmid 11954) were prepared [35] and kindly provided by Dr. Nico Dantuma of the Karolinska Institutet, Sweden. Plasmids expressing ULK1 (Addgene plasmid 31961) were prepared [36] and kindly provided by Dr. Do-Hyung Kim of the University of Minnesota.

### Cell culture

Human umbilical vein endothelial cells (HUVECs), human embryonic kidney cell line 293 (HEK293), and GFPu-1 cells, as well as their mediums, were obtained from ATCC (Manassas, VA). U20S cells expressing the GFP-LC reporter were procured from EMD Millipore (Billerica, MA). HUVECs were cultured in EBM containing 5% fetal bovine serum (FBS). We used 4–6 passages of HUVECs, which reached 80% confluence for the experiments. Mouse embryonic fibroblasts (MEF), HEK293, U20S, and GFPu-1 cells were grown in DMEM with 10% FBS and penicillin (100 u/ml)/streptomycin (100 µg/ml). All of these cells were cultured in a cell incubator at a humidified atmosphere of 5% CO2+95% O2 at 37°C.

### Western blot analysis

Cultured cells or mouse tissues were homogenized on ice in 1× cell-lysis buffer. Protein concentrations were determined by the BCA protocol. Proteins were separated on PAGE gel (8–12%) and transferred to the PVDF membranes. The membranes were blocked for 2 hours in 5% non-fat milk (wt/vol.) and incubated with primary antibodies overnight at 4°C. The next day, they were incubated with horseradish peroxidase-conjugated rabbit anti-mouse (1∶10000) or goat anti-rabbit IgG (1∶10000) for 2 hours at room temperature. The bands were identified using the standard chemiluminescence method. Band intensity was quantified with Photoshop software.

### RT-PCR

Total cellular RNA was isolated from HUVECs and HEK293 cells using the Total RNA Kit I (#R683401) purchased from Omega Bio-Tek (Norcross, GA). The cDNA was synthesized using the iScriptTM cDNA Synthesis Kit (170–8891, Bio-Rad). The resulting cDNA was subjected to quantitative polymerase chain reaction (PCR) using the iQTM SYBR Green Supermix (170–8880). The Real Time Detection System was obtained from Bio-Rad (Hercules, CA). SIRT1 primer (Forward: 5′-GCAGGTTGCGGGAATCCAA-3′ and reverse: 5′-GGCAAGATGCTGTTGCAAA-3′) and 18 s primer (Forward: 5′-TGCTGCAGTTAAAAAGCTCGT-3′ and reverse: 5′-GGCCTGCTTTGAACACTCTAA-3′) were synthesized by Sigma (St. Louis, MO).

### Detection of O-GlcNAc-modified proteins in cell lysates and mouse aortic homogenates

Cell lysates were prepared in lysis buffer which contained 25 mM HEPES (pH 7.0), 1 mM EDTA, 1 mM EGTA, 1% NP-40, 0.1% SDS, 1% protease inhibitor cocktail (Sigma, St Louis, MO), 1% phosphatase inhibitor cocktail, and 100 µM PUGNAc (O-GlcNAcase inhibitor). Agarose bound Wheat Germ Agglutinin Kit (WGA) was used to pull down proteins modified by O-linked GlcNAc according to instructions of the manufacturer, Vectorlabs (Burlingame, CA), and as described previously [37].

### Determination of 26S proteasome activity

26S proteasome activity was determined by one of its protease-like activities, as previously described with minor modifications [38], [39]. The chymotrypsin-like activity was measured using a fluorogenic proteasome substrate SucLLVY-7-amido-4-methylcoumarin (AMC) at a final concentration of 80 µM in 1% dimethyl sulfoxide (DMSO). ATP-dependent cleavage activity was monitored continuously by detection of free 7-amido-4-methylcoumarin with a Fluorescence Microplate Reader (Wallach Victor from PerkinElmer, Waltham, MA) at 380/460 nm at 37°C.

### Mouse embryonic fibroblasts (MEF)

The immortalized mouse embryonic fibroblasts (MEF) made from *Ulk1* ^−/−^, *Ulk2* ^−/−^, *Ulk1^−/−^/2^−/−^*, and wild-type mice were obtained from Dr. Sharon Tooze of Cancer Research UK, who isolated and prepared these cell lines as previously described [40], [41].

### Transfection with adenovirus, plasmid, and siRNA duplex

Transfection with adenovirus encoding eNOS, OGT, or GFP (as adenoviral infection control) was performed as described previously [27], [32], [33]. Transfection with plasmid encoding Ub^G76V^-GFP was conducted as previously reported [35]. Transfection of control or target siRNA was performed based on protocols provided by Santa Cruz Biotechnology (Santa Cruz, CA), as described previously [42].

### Mice

Male *eNOS^−/−^* mice (10 weeks), db/db mice (25 weeks), and the age-matched C57BL/6J WT mice were originally obtained from the Jackson Laboratory (Bar Harbor, ME) at younger ages. Animals were housed under controlled temperature (21°C) and cyclic lighting, with 12 hours of light and 12 hours of dark. All mice had free access to water and a standard mouse chow diet. Mice were handled in accordance with the protocols reviewed and approved by the Institutional Animal Care and Use Committee of the University of Oklahoma Health Sciences Center (Oklahoma City, OK). The mice were sacrificed by CO_2_-induced euthanasia for tissue collection. The CO_2_-induced euthanasia was performed by placing mice in a container and exposing them to a gradually increasing concentration of CO_2_. The mice ceased breathing within 30 sec and were left in the CO_2_ atmosphere for a total of 5 minutes. The investigation conformed to the Guide for the Care and Use of Laboratory Animals published by the United States National Institutes of Health.

### Statistical analysis

Data were reported as mean ±*SEM*. ANOVA was used to compare means of different experimental groups. Tukey's Tests were used as post-hoc tests. A *p* value <0.05 was considered statistically significant.

## Results

### eNOS stabilizes and increases SIRT1 protein expression in vascular endothelial cells

**Figure 1:**
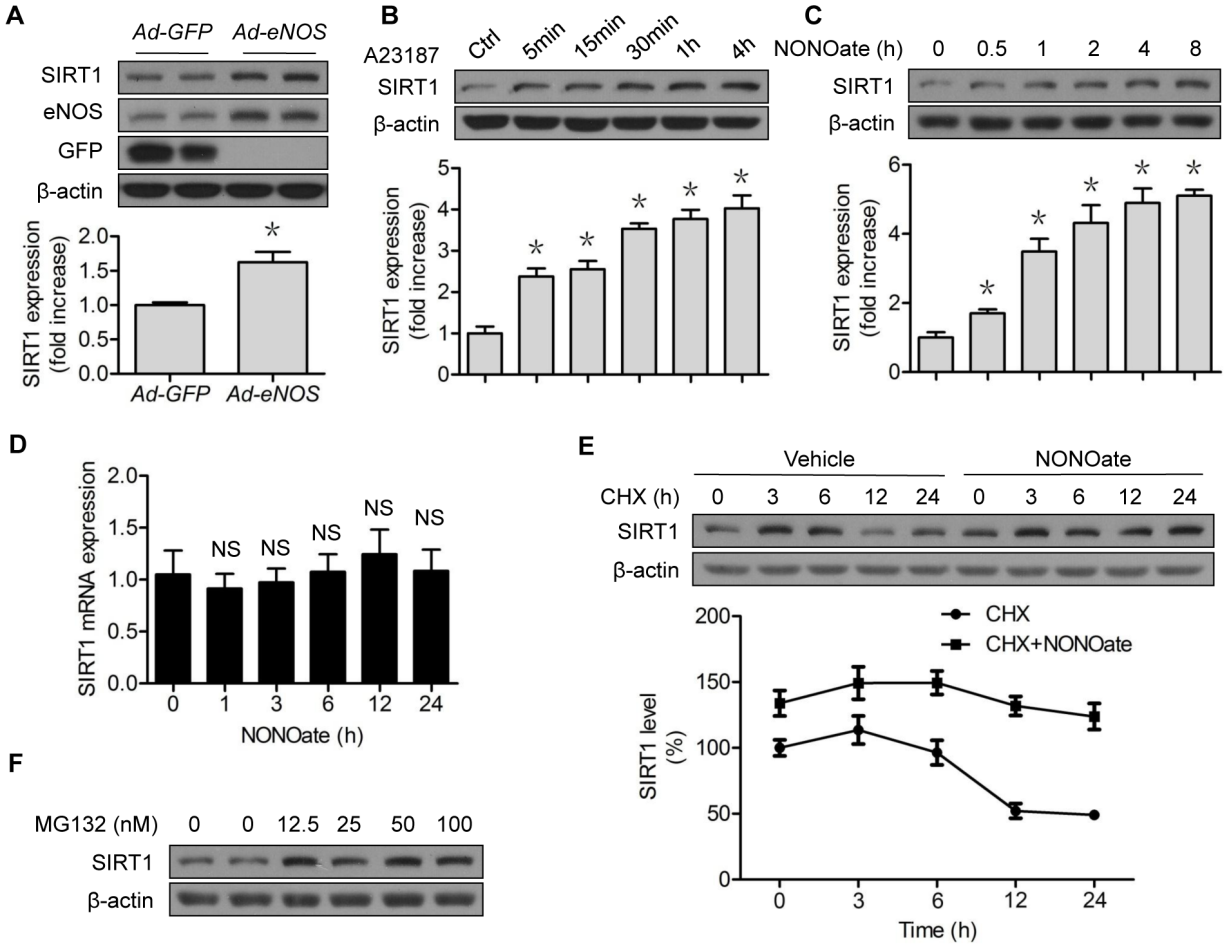
NO stabilizes and increases SIRT1 protein expression. (A) HUVECs were transfected with GFP or eNOS adenovirus for 48 h; (B) HUVECs were treated with A23187 (1 µM) for the indicated time; (C) HUVECs were treated with NONOate (50 µM) for the indicated time; (D) HUVECs were treated with NONOate (50 µM) for the indicated time; SIRT1 mRNA levels were determined by RT-PCR; (E) HUVECs were treated with CHX (5 µM) for the indicated time, followed by incubation of NONOate (50 µM) for 4 h; (F) HUVECs were treated with the indicated concentrations of MG132 for 6 h. The western blots are representative of three independent experiments. *represents *p*<0.05 vs control (*n* = 3); NS, not significant. GFP, green fluorescent protein; eNOS, endothelial nitric oxide synthase; Ad, Adenovirus; CHX, cycloheximide.

To confirm that eNOS positively regulates SIRT1, we performed adenoviral overexpression of eNOS in HUVECs. Increased eNOS protein expression was associated with SIRT1 protein upregulation (Fig. 1A). The upregulation was recapitulated in a time-dependent fashion, when the control cells were pretreated with A23187, an eNOS activator (Fig. 1B), or with DETA-NONOate (Diethylenetriamine NONOate), an NO donor that is widely used in cell studies (Fig. 1C), suggesting NO regulation of SIRT1. However, NONOate did not significantly increase SIRT1 mRNA (Fig. 1D), suggesting that a post-translational mechanism, such as protein stability, was involved. A post-translational mechanism is suggested because SIRT1 can be ubiquitinated for proteasomal degradation [15], and NO blocks 26S proteasome functionality [27]. To test this, we performed chase experiments with cycloheximide (CHX), a blocker of translational elongation. As expected, CHX alone reduced SIRT1 protein expression in a time-dependent fashion (Fig. 1E). In the presence of NONOate, SIRT1 protein stability was significantly increased (Fig. 1E). In addition, incubation of HUVECs with MG132, a potent 26S proteasome inhibitor, presented a dose-dependent upregulation of SIRT1 (Fig. 1F). These data suggest that the proteolysis mediated NO regulation of SIRT1 turnover, likely through the proteasome.

### NO stabilizes and increases ULK1 protein expression in vascular endothelial cells

**Figure 2:**
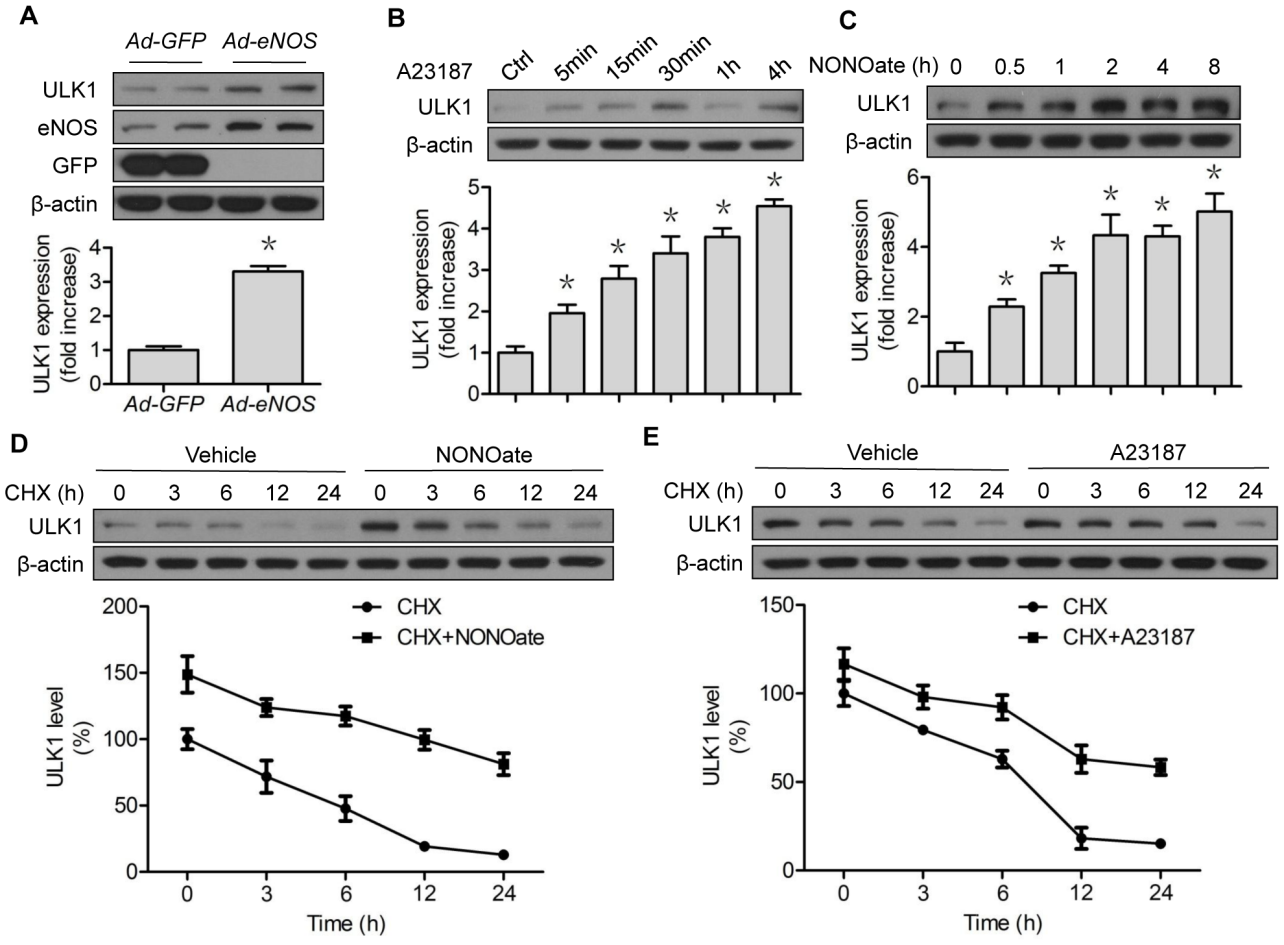
NO stabilizes and upregulates ULK1 protein expression. (A) HUVECs were transfected with GFP or eNOS adenovirus for 48 h; (B) HUVECs were treated with A23187 (1 µM) for the indicated time; (C) HUVECs were treated with NONOate (50 µM) for the indicated time; (D) HUVECs were treated with CHX (5 µM) for the indicated time, followed by incubation of NONOate (50 µM) for 4 h; (E) HUVECs were treated with CHX (5 µM) for the indicated time, followed by incubation of A23187 (1 µM) for 4 h. The western blots are representative of three independent experiments. *represents *p*<0.05 vs control (*n* = 3); NS, not significant. GFP, green fluorescent protein; eNOS, endothelial nitric oxide synthase; Ad, Adenovirus; CHX, cycloheximide.

Recent work from our laboratory [43] and others [44] supports the notion that redox signaling controls autophagy, another important and conserved pathway that maintains cellular proteostasis [45]. NO has been reported to inhibit autophagy [46], although discordant observations have been made, depending on cell type and assay condition [47]. We tested whether NO altered unc-51-like kinase (ULK1), an important autophagy-related protein, by initiating the formation of autophagosome [48]. Surprisingly, overexpression of eNOS in HUVECs upregulated ULK protein levels (Fig. 2A). This effect was reproduced by administration of an eNOS activator (A23187, Fig. 2B) or an NO donor (NONOate, Fig. 2C), in a time-dependent manner. In chase experiments, both NONOate (Fig. 2D) and A23187 (Fig. 2E) markedly prolonged SIRT1 protein stability in the presence of CHX. Since ULK1 could be a proteasome substrate [49], these results indicated that NO also regulated ULK1 protein stability.

### NO does not restore SIRT1 protein expression when ULK1 is downregulated

**Figure 3:**
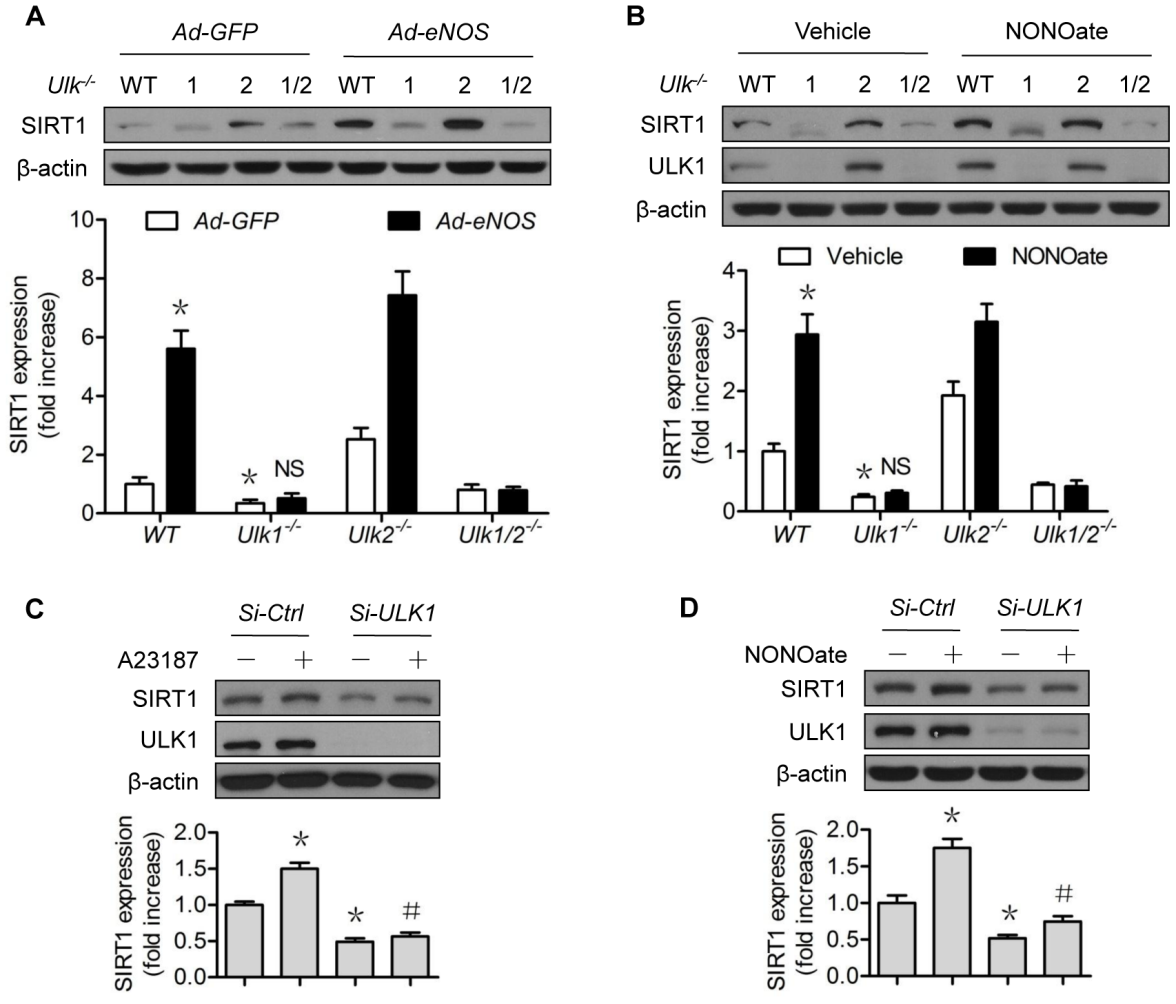
ULK1 mediates the NO-induced SIRT1 upregulation. (A) MEF from the *Ulk1* ^−/−^, *Ulk2* ^−/−^, *Ulk1/2* ^−/−^, and *WT* mice were transfected with GFP or eNOS Adenovirus for 48 h; (B) MEF from the *Ulk1* ^−/−^, *Ulk2* ^−/−^, *Ulk1/2* ^−/−^, and *WT* mice were treated with NONOate (50 µM) or A23187 (1 µM) for 4 h; (C) HUVECs were transfected with control or *ULK1* SiRNA for 48 h, then treated with A23187 (1 µM) for 4 h; (D) HUVECs were transfected with control or *ULK1* SiRNA for 48 h, then treated with NONOate (50 µM) for 4 h. The western blots shown are representative of three independent experiments. *represents *p*<0.05 vs control (*n* = 3); #represents *p*<0.05 vs A23187 or NONOate alone (*n* = 3); NS, not significant. GFP, green fluorescent protein; eNOS, endothelial nitric oxide synthase; Ad, Adenovirus; Si-SiRNA.

ULK1 appeared to have a shorter half-life than that of SIRT1 (Fig. 1E *vs* Fig. 2D). We wondered whether ULK1 was required in the NO regulation of SIRT1. Overexpression of human eNOS upregulated SIRT1 protein expression in *WT* and *Ulk2^−/−^* MEF, but this did not occur in *Ulk1* ^−/−^ and *Ulk1/2* ^−/−^ MEF (Fig. 3A). Administration of NONOate produced similar results (Fig. 3B), indicating that the ULK1 isoform mediated the NO-exerted effects. To confirm the role of ULK1 in vascular endothelial cells, we performed siRNA knockdown experiments in HUVECs. A23187 (Fig 3C) and NONOate (Fig 3D) increased the SIRT1 protein levels in control siRNA-treated cells, but failed to do so in the *ULK1*-siRNA treated cells (Fig. 3C and 3D). These data suggest that the NO-ULK1-SIRT1 axis might be operative in cells.

### ULK1 regulates SIRT1 protein expression in vascular endothelial cells

**Figure 4:**
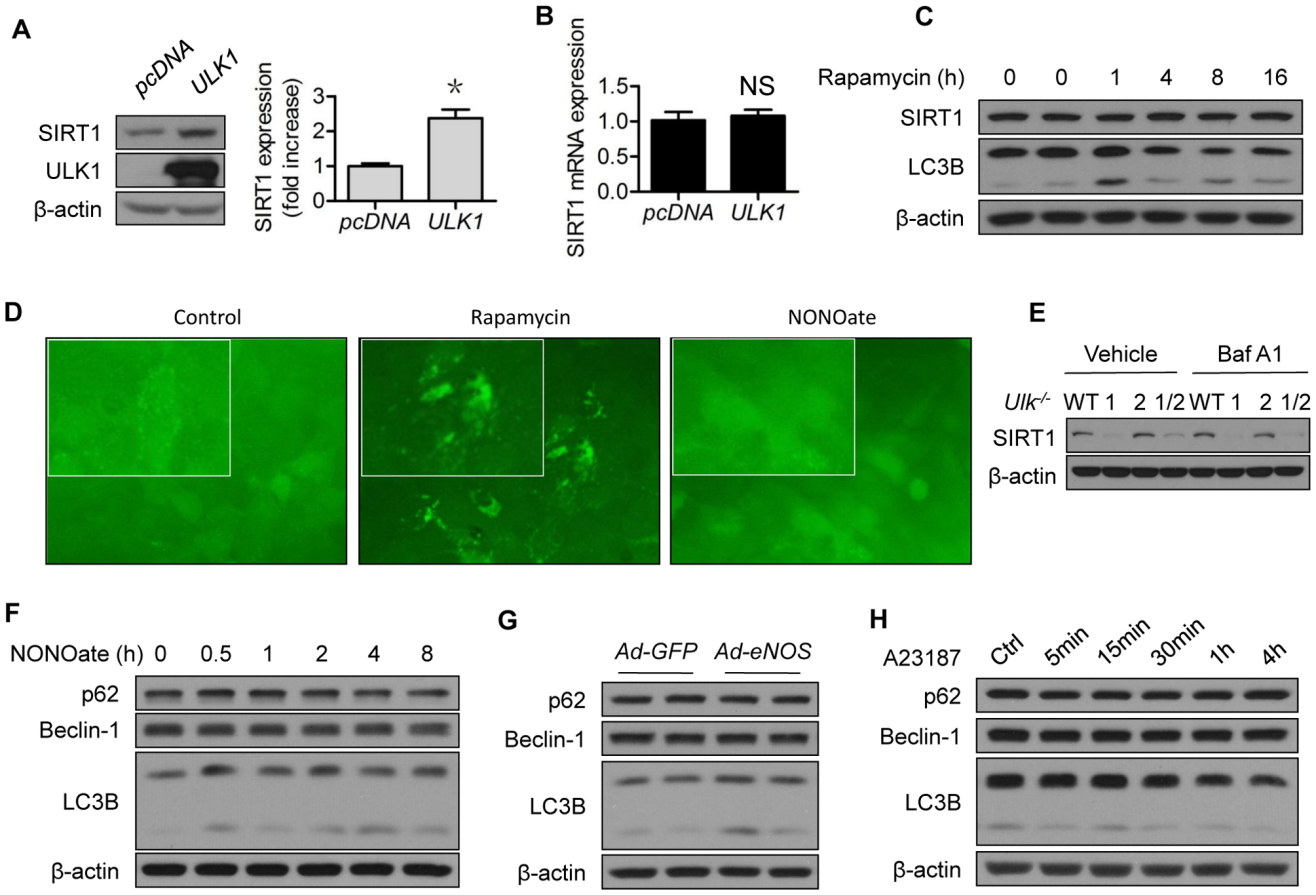
ULK1 regulates SIRT1 protein expression which is independent of autophagy. (A) HEK293 cells were transfected with *pcDNA* or *ULK1* plasmid for 48 h; (B) HEK293 cells were transfected with *pcDNA* or *ULK1* plasmid for 48 h. SIRT1 mRNA levels were determined by RT-PCR; (C) HUVECs were treated with rapamycin (2 µM) for the indicated time; (D) U20S cells which expressed GFP-LC3 reporter were treated with rapamycin (2 µM) or NONOate (50 µM) for 12 h; GFP-LC3 imaging was captured using fluorescent microscope according to instructions of the manufacturer, EMD Millipore; (E) MEF from the *Ulk1* ^−/−^, *Ulk2* ^−/−^, *Ulk1/2* ^−/−^, and *WT* mice were treated with bafilomycin A1 (10 nM) for 4 h; (F) HUVECs were treated with NONOate (50 µM) for the indicated time; (G) HUVECs were transfected with GFP or eNOS adenovirus for 48 h; (H) HUVECs were treated with A23187 (1 µM) for the indicated time. The western blots are representative of three independent experiments. *represent *p*<0.05 vs control (*n* = 3). NS, not significant. GFP, green fluorescent protein; eNOS, endothelial nitric oxide synthase; Ad, adenovirus; Baf A1, Bafilomycin A1.

The possibility for ULK1 regulation of SIRT1 was partly supported by the loss-of-function approach, in which the *Ulk1^−/−^* MEF alone presented lower SIRT1 abundance than *WT* MEF (Fig. 3A and 3B). Similarly, lower SIRT1 protein expression was confirmed in *ULK1*-siRNA- than in control-siRNA-treated HUVECs (Fig. 3C and 3D). We took a gain-of-function approach to further determine the relationship between ULK1 and SIRT1. As shown in Fig. 4, transient expression of *ULK1* plasmid increased SIRT1 protein levels (Fig. 4A), without altering *SIRT1* mRNA levels (Fig. 4B).

### Modulation of autophagy does not mimic the regulation of SIRT1 by ULK1 or NO

ULK1 is known to mediate starvation-induced autophagy by initiating the formation of autophagosome [48]. To determine whether autophagy induction would mimic the effect of ULK1 upregulation on SIRT1, we treated the cells with rapamycin, a well-recognized inducer of autophagy that functions by inhibiting mTOR. Rapamycin induced autophagy marker LC3B (Fig. 4C) and autophagic flux (Fig. 4D), but did not increase SIRT1 protein expression (Fig. 4C). To determine whether autophagy mediated the SIRT1 downregulation in *Ulk1^−/−^* MEF, we incubated the MEF with Baf A1, an autophagy inhibitor which blocks the fusion between autophagosomes and lysosomes. As shown, Baf A1 did not restore SIRT1 protein expression (Fig. 4E). The effects of NONOate on autophagic flux were minimal (Fig. 4D). The impact of NONOate on common autophagy markers was not significant (Fig. 4F), similar to the effects of eNOS (Fig. 4G) and A23187 (Fig. 4H). Taken together, these data suggest that regulation of ULK1 turnover by ULK1 or NO occurs independent of autophagy.

### ULK1 depletion induces proteasomal degradation of SIRT1

**Figure 5:**
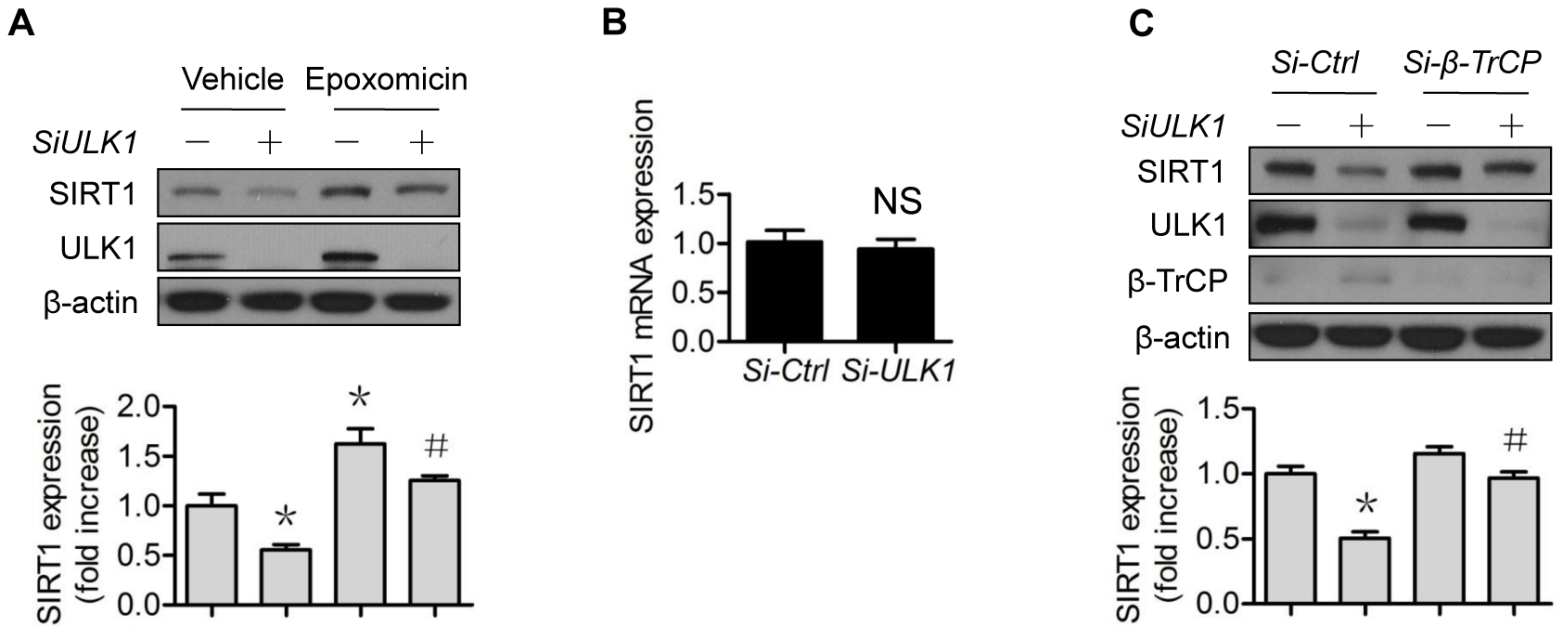
ULK1 regulates SIRT1 protein expression via 26S proteasomes. (A) HUVECs were transfected with control or *ULK1* SiRNA for 48 h, then treated with epoxomicin (0.1 µM) for 4 h; (B) HUVECs were transfected with control or *ULK1* siRNA for 48 h, SIRT1 mRNA levels were determined by RT-PCR; (C) HUVECs were transfected with control or *ULK1* or β-TrCP1 siRNA for 48 h. The western blots are representative of three independent experiments. *represents *p*<0.05 vs control (*n* = 3); # represents *p*<0.05 vs *si-ULK1* alone (*n* = 3). Si, siRNA.

To confirm the involvement of 26S proteasomes in the ULK1 regulation of SIRT1, we compared SIRT1 protein expression in *ULK1* siRNA-treated cells, with or without 26S proteasome inhibition. In the presence of epoxomicin, a potent proteasome inhibitor, SIRT1 protein expression was significantly restored in *ULK1*-siRNA-treated cells (Fig. 5A). Since *ULK1*-siRNA treatment did not change *SIRT1* mRNA levels (Fig. 5B), these data demonstrate that ULK1 depletion induced proteasomal degradation of SIRT1.

### β-TrCP1 mediates proteasomal degradation of SIRT1 induced by ULK1 depletion

Most proteasome-mediated protein degradations require a specific ubiquitin E3 ligase complex. To further confirm the proteasomal degradation of SIRT1, we sought to determine the responsible ubiquitin E3 ligase. As shown, siRNA knockdown of β-TrCP1reversed SIRT1 protein expression induced by *ULK1*-siRNA treatment, while knockdown of control siRNA did not have the same effect (Fig. 5C).

### ULK1 regulates 26S proteasome functionality

**Figure 6:**
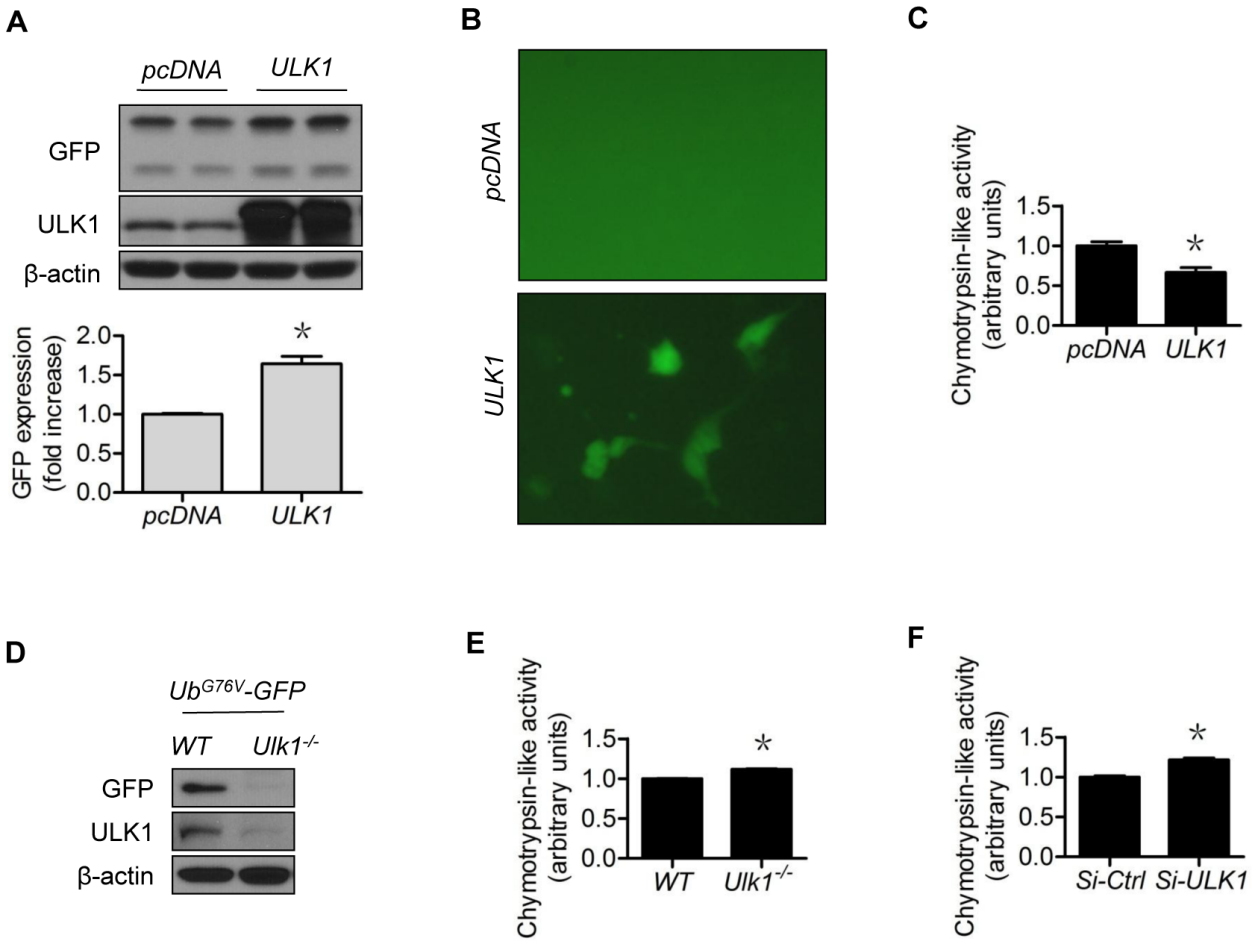
ULK1 modulates 26S proteasome functionality. GFPu-1 cells were transfected with (A) control or *ULK1* plasmid for 48 h, and (B) the accumulated GFP fluorescence in *ULK1* plasmid-expressing cells was captured with a fluorescent microscope; (C) HEK293 cells were transfected with control or *ULK1* plasmid for 48 h and chymotrypsin-like activity was measured; (D) The *Ulk1* ^−/−^ and *WT* MEF were transfected with Ub^G76V^-GFP plasmid for 48 h; (E) Chymotrypsin-like activity was measured in the *Ulk1* ^−/−^ and *WT* MEF; (F) HUVECs were transfected with control or *ULK1* SiRNA for 48 h and chymotrypsin-like activity was measured. The western blots are representative of three independent experiments. *represents *p*<0.05 vs control (*n* = 3). Si, siRNA.

We wondered if the ULK1 regulation of SIRT1 was achieved via regulation of 26S proteasome functionality. To test this possibility, we used GFPu-1 cells, a cell line derived from HEK293 cells stably expressing the GFP-CL1 degron as the proteasome reporter [50]. Compared with control plasmid (vector) transfected GFPu-1 cells, the upregulation of ULK1 in *ULK1*-plasmid-treated cells increased both GFP protein levels (Fig. 6A) and GFP fluorescence (Fig. 6B), indicating 26S proteasome suppression. These changes were accompanied by reduced 26S proteasome activity, evidenced by decreased chymotrypsin-like protease activity (Fig. 6C). The data showed that ULK1 upregulation blocked the 26S proteasome functionality. To determine if lack of ULK1 could do the opposite, we treated the *Ulk1* ^−/−^ MEF with plasmids expressing Ub^G76V^-GFP, a 26S proteasome reporter similar to GFPu-1, but using ubiquitin fusion degradation as the degron (Ub^G76V^-GFP) [35]. As demonstrated, the *Ulk1* ^−/−^ MEF presented significantly less GFP protein compared with *WT* MEF (Fig. 6D). Loss of *Ulk1* was associated with slightly increased proteasome activity (Fig. 6E), similar to the *ULK1*-siRNA-treated HUVECs (Fig. 6F). Together, these data indicate that modulation of ULK1 changed 26S proteasome functionality.

### ULK1 inhibits 26S proteasome functionality via OGT

**Figure 7:**
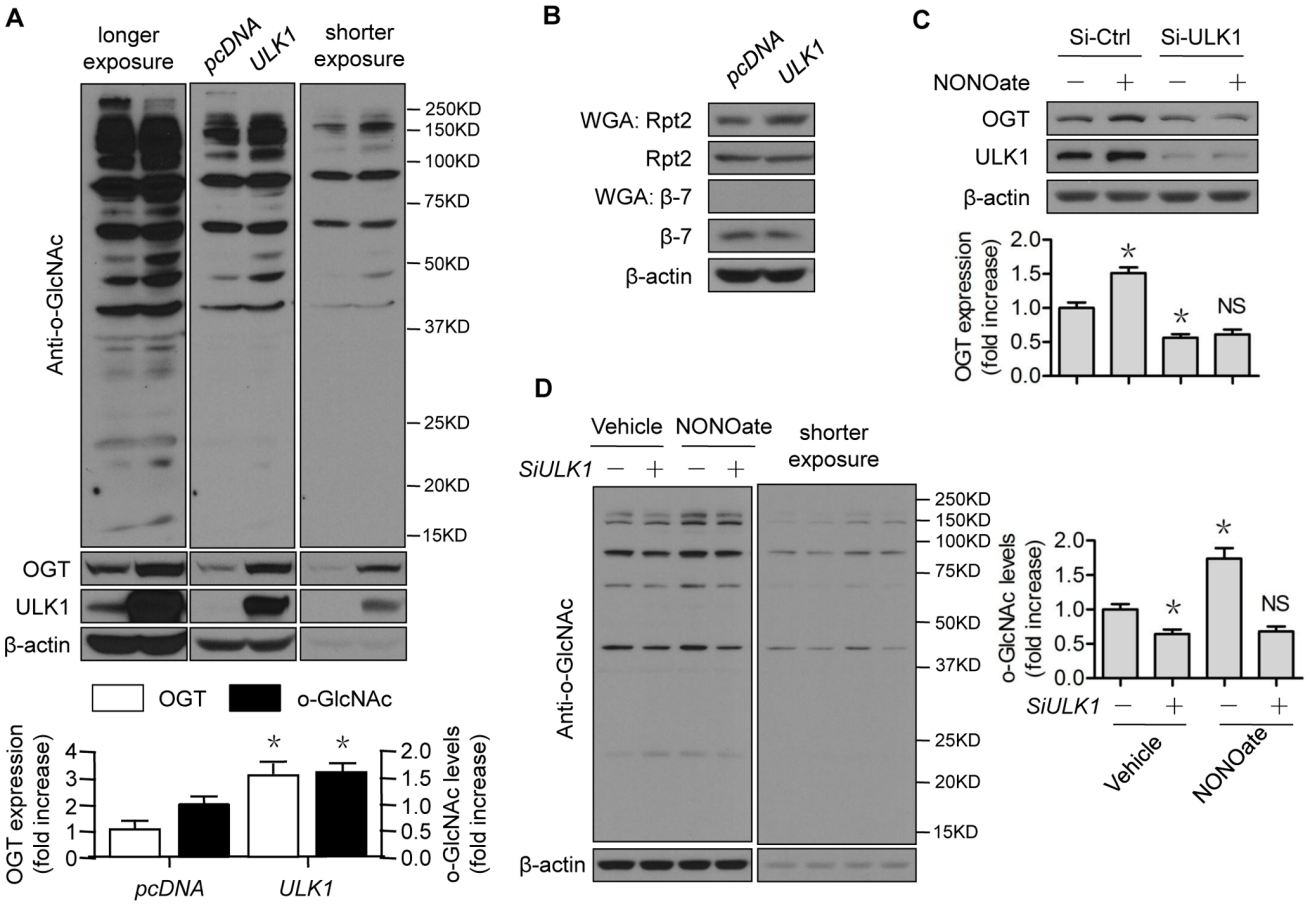
ULK1 regulates 26S proteasome functionality via OGT. (A–B) HEK293 cells were transfected with control or *ULK1* plasmid for 48 h; (C–D) HUVECs were transfected with control or *ULK1* siRNA for 48 h, then treated with NONOate (50 µM) for 4 h. The western blots are representative of three independent experiments. *represents *p*<0.05 vs control (*n* = 3); NS, not significant vs *si-ULK1* alone. OGT, O-GlcNAc transferase; WGA, wheat germ agglutinin; Si, siRNA.

We previously demonstrated that NO negatively regulates 26S proteasome functionality through an OGT-dependent pathway [27]. We questioned whether ULK1 would mediate this pathway. Overexpression of ULK1 significantly upregulated OGT protein expression (Fig. 7A). The upregulation was accompanied by elevated levels of O-GlcNAc-modified protein, as probed with an anti-GlcNAc antibody (Fig. 7A). We used a WGA Kit [37] to enrich O-GlcNAc-modified proteins and detected the increased O-GlcNAcylation of Rpt2 (Fig. 7B), a key component of the regulatory complex (19S) of the 26S proteasome and an ATPase [51], O-GlcNAc modification of which induced proteasome suppression [51]. While the inputs of selected proteasome subunits, such as β7, were unchanged, the O-GlcNAc modification of β7 was undetectable (Fig. 7B), suggesting potential substrate specificity for O-GlcNAc modification. In contrast, downregulation of ULK1 by siRNA treatment decreased OGT protein expression (Fig. 7C) and O-GlcNAc-modified protein levels (Fig. 7D). Confirming our previous study [27], NONOate increased the levels of OGT and O-GlcNAc modified proteins in control-siRNA-treated cells. However, these effects were compromised in *ULK1*-siRNA treated cells (Fig. 7C and 7D). These results strongly suggest that the ULK1 modulation of OGT regulated the NO-induced 26S proteasome blockage.

### The NO-ULK1-SIRT1 axis seems operative in eNOS-knockout mice and db/db mice

**Figure 8:**
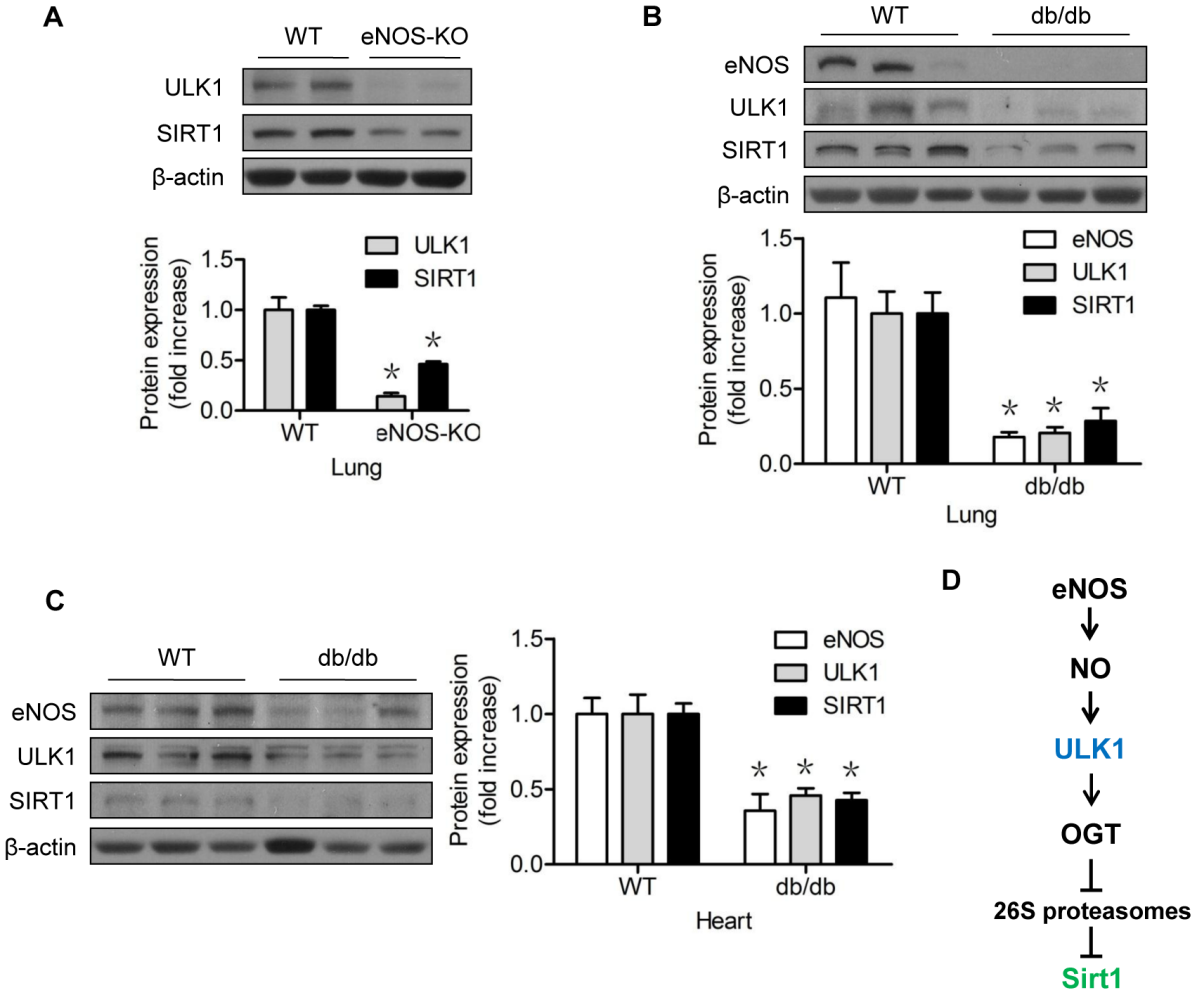
The NO-ULK1-SIRT1 pathway might be operative in the whole animal. (A) Western blots of lung tissues of wild-type (C57BL/6J) and eNOS knock-out mice; (B) Western blots of lung tissues of wild-type (C57BL/6J) and db/db mice; (C) Western blots of lung tissues of wild-type (C57BL/6J) and db/db mice; (D) The proposed mechanism of the eNOS-derived NO regulation of SIRT1 protein turnover, which requires ULK1 and OGT, in vascular endothelial cells. *represents *p*<0.05 vs WT (*n* = 3 in eNOS-KO model; *n* = 5 in db/db model). WT, wild-type; eNOS, endothelial nitric oxide synthase; OGT, O-GlcNAc transferase.

To confirm that the proposed NO-ULK1-SIRT1 pathway was operative in the whole animal, we monitored the ULK1 and SIRT1 protein levels in tissues obtained from two mouse models. In the lungs of eNOS-KO mice, the genotype of which has been confirmed in our previous studies [27], the absence of eNOS was associated with the downregulation of ULK1 and Sirt1 proteins (Fig. 8A). In type 2 diabetic db/db mice, eNOS protein expression was reduced in the lungs (Fig. 8B) and hearts (Fig. 8C). This reduction was correlated with a significant reduction of ULK1 and SIRT1 protein expression (Fig. 8B and 8C).

## Discussion

In the present study, we identified a new pathway for NO regulation of SIRT1 (Fig 8D). We found that NO stabilized and upregulated SIRT1 protein expression through an ULK1-dependent pathway. The expression of ULK1 could be stabilized by NO. Mechanistically, ULK1 negatively regulated 26S proteasome functionality via an OGT-dependent mechanism, leading to stabilization and upregulation of SIRT1. Conversely, downregulation of ULK1 enhanced 26S proteasome functionality, leading to accelerated degradation of SIRT1, mediated by the ubiquitin E3 ligase β-TrCP1. Given the central role of SIRT1 in extending the lifespan and improving vascular health, these findings will enhance our recognition of the fundamental importance of NO-regulated 26S proteasome functionality to vascular homeostasis.

The physiological regulation of 26S proteasomes is complex and operates through mechanisms that are incompletely understood. Multiple mechanisms are believed to be involved, including post-translational modifications [52]. O-GlcNAc modification was the first identified endogenous inhibitor of the 26S proteasome [51], [52]. As an ATPase and a key component of the regulatory sub-complex of 26S proteasomes, Rpt2 is modified by O-GlcNAc *in vitro* and *in vivo*. Interestingly, when Rpt2 modification increases, the 26S proteasome function decreases, by a mechanism involving ATPase and OGT [51]. Hence, O-GlcNAc modification is considered an endogenous inhibitor of the 26S proteasome [51], and O-GlcNAc modification connects a nutritional sensor to proteasome functional regulation [53]. By utilizing the 26S proteasome reporter (Ub^G76V^-GFP) system in both cultured cells and mice, we have recently identified NO, particularly eNOS-derived NO, as an endogenous regulator of the 26S proteasome in vascular endothelial cells. Mechanistically, NO upregulates this intrinsic proteasome inhibitory pathway, leading to suppression of the 26S proteasome functionality in vascular endothelial cells [27].

Until now, the mechanism by which NO positively regulated OGT was unknown. Moreover, no functional substrate(s) had been connected to this mechanism. As a logical extension of our previous study [27], the present study has answered these questions. First, we demonstrated that ULK1 was the mechanism underlying NO regulation of 26S proteasome functionality. Loss of ULK1 attenuated the impacts of NO on the protein stability of the proteasome substrate SIRT1 (Fig. 3), as well as O-GlcNAc modification (Fig. 7D). Modulation of ULK1 also regulated levels of OGT (Fig. 7A), GlcNAcylation (Fig. 7A and 7D), and 26S proteasome functionality (Fig. 6), mimicking NO-elicited impacts. It is likely that the NO-ULK1-SIRT1 axis operated in the whole animal (Fig 8A, 8B, and 8C), based on our data obtained from cell studies.

Then, we identified SIRT1 as the functional target for NO-regulated 26S proteasome functionality. Identification of this connection is significant. Strong *in vitro* and *in vivo* evidence supports the positive regulation of SIRT1 by NO [21], [22], [23], [24], [25], [26], but the mechanistic details of the regulation were unclear. The mechanism for SIRT1 protein turnover regulation was also poorly defined, in contrast to the extensive investigations that have focused on identifying the cellular targets and functional networks controlled by SIRT1.

The mechanisms underpinning the biological regulation of SIRT1 activity have only recently begun to emerge [54]. The pleiotropic effects of SIRT1 stem from the network SIRT1 controls through its enzymatic activity. SIRT1 exclusively uses NAD^+^ as a co-substrate, so the regulation of SIRT1 activity through NAD^+^ is well established. Post-translational modifications (PTMs) of SIRT1 are the common forms that regulate enzyme activity. JNK phosphorylates SIRT1 at Ser27 and 47, and Thr530, particularly under stressful cellular conditions. These modifications increase the deacetylase activity of SIRT1 towards histone H3, but have no effect on p53, although both are SIRT1 substrates. This suggests that JNK PTMs are substrate-specific [55]. Additional kinases, such as CDK1, casein kinase (CK)2, and PKA have been shown to induce SIRT1 phosphorylation. Other forms of PTMs have also been reported, such as methylation [56], SUMOylation [57], and nitrosylation [58].

A growing list of transcription factors, including CREB, ChREBP, FOXO1, FOXO3, and PPARs, modulate SIRT1 activity by changing its expression levels, and particularly its regulation at transcriptional levels [59]. The abundance of SIRT1 is also controlled by post-transcriptional events, such as RNA stability. The best example of this occurs though Hu antigen R (HuR). The half-life of *SIRT1* mRNA drastically declines in the absence of HuR, leading to lower SIRT1 expression and activity [60]. Proteasomal degradation has been recently implicated, as ubiquitination of SIRT1 targeting for degradation has been detected [15], although the responsible ubiquitin E3 ligase was not identified.

In the present study, we first confirmed that the 26S proteasomes contributed to loss of SIRT1 stability. NO likely increased protein stability of SIRT1 by suppressing its proteasomal degradation (Fig. 1E), because NO did not alter *SIRT1* mRNA levels in the studied settings (Fig. 1D), whereas proteasome suppression upregulated SIRT1 protein levels (Fig. 1F). However, depletion or lack of ULK1 compromised the effects of NO on SIRT1 protein stability, suggesting the involvement of ULK1 (Fig. 3). This notion was further supported by several lines of evidence demonstrating proteasomal degradation of SIRT1 induced by downregulation of ULK1 (Fig. 5A), the capacity of ULK1 in modulating 26S proteasome functionality (Fig. 6), and the identification of the responsible ubiquitin E3 ligase (Fig. 5C). To the best of our knowledge, this is the first evidence of ubiquitin E3 ligase being responsible for ULK1 proteasomal degradation, consistent with the interaction between SIRT1 and β-TrCP1 that has been reported by others [61]. Interestingly, the reported interaction actually determines the turnover of β-TrCP1. While upregulation of SIRT1 accelerates proteasomal degradation of β-TrCP1, downregulation of SIRT1 stabilizes β-TrCP1 [61]. The latter was reproduced in our study, as SIRT1 downregulation by *ULK1*-siRNA treatment was associated with increased β-TrCP1 (Fig. 5C). Whether the expression ratio of SIRT1/β-TrCP1 is crucial to its own stability merits further investigation.

Clearly, modulation of ULK1 tuned SIRT1 expression levels (Fig. 3; Fig. 4A; Fig. 5A and 5C). ULK1 could be a substrate of 26S proteasomes, as shown in HFD-fed mice [62]. Therefore, NO-induced ULK1 augmentation (Fig. 2) and consequently SIRT1 upregulation (Fig. 1) could be fully presented by ULK1 overexpression. ULK1 overexpression led to increased accumulation of SIRT1 protein (Fig. 4A) and proteasome reporter protein, an indication of 26S proteasome suppression (Fig. 6A and 6C). Although ULK1-mediated NO regulation of SIRT1 appeared to be autophagy-independent, the possibility that NO affects autophagy, dependent on or independent of ULK1, leading to turnover of other proteins cannot be excluded. In any case, ULK1-mediated NO regulation of proteasomal degradation provides a novel and viable avenue to regulate SIRT1 activity.

Collectively, the findings from the present study identified a new mechanism for NO regulation of SIRT1. This mechanism requires modulation of the 26S proteasome, and the involvement of ULK1, an autophagy-related protein and OGT, an enzyme that regulates O-GlcNAcylation. These findings should advance our understanding of the connection between NO bioavailability and the beneficial effect of SIRT1 in endothelial cells. Our study identifies a potential route for therapeutic modulation of SIRT1 protein levels in SIRT1-linked diseases including cancer, neurodegeneration, and diabetes.
